# Genomic Stability over 9 Years of an Isoniazid Resistant *Mycobacterium tuberculosis* Outbreak Strain in Sweden

**DOI:** 10.1371/journal.pone.0016647

**Published:** 2011-01-31

**Authors:** Linus Sandegren, Ramona Groenheit, Tuija Koivula, Solomon Ghebremichael, Abdolreza Advani, Elsie Castro, Alexandra Pennhag, Sven Hoffner, Jolanta Mazurek, Andrzej Pawlowski, Boris Kan, Judith Bruchfeld, Öjar Melefors, Gunilla Källenius

**Affiliations:** 1 Department of Medical Biochemistry and Microbiology, Uppsala University, Uppsala, Sweden; 2 Swedish Institute for Infectious Disease Control, Solna, Sweden; 3 Department of Microbiology, Tumor and Cell Biology, Karolinska Institutet, Stockholm, Sweden; 4 Department of Clinical Science and Education, Karolinska Institutet, Stockholm, Sweden; 5 Infectious Diseases Unit, Department of Medicine, Karolinska Institutet, Karolinska University Hospital, Solna, Sweden; Fundació Institut Germans Trias i Pujol; Universitat Autònoma de Barcelona CibeRES, Spain

## Abstract

In molecular epidemiological studies of drug resistant *Mycobacterium tuberculosis* (TB) in Sweden a large outbreak of an isoniazid resistant strain was identified, involving 115 patients, mainly from the Horn of Africa. During the outbreak period, the genomic pattern of the outbreak strain has stayed virtually unchanged with regard to drug resistance, IS*6110* restriction fragment length polymorphism and spoligotyping patterns. Here we present the complete genome sequence analyses of the index isolate and two isolates sampled nine years after the index case as well as experimental data on the virulence of this outbreak strain. Even though the strain has been present in the community for nine years and passaged between patients at least five times in-between the isolates, we only found four single nucleotide polymorphisms in one of the later isolates and a small (4 amino acids) deletion in the other compared to the index isolate. In contrast to many other evolutionarily successful outbreak lineages (e.g. the Beijing lineage) this outbreak strain appears to be genetically very stable yet evolutionarily successful in a low endemic country such as Sweden. These findings further illustrate that the rate of genomic variation in TB can be highly strain dependent, something that can have important implications for epidemiological studies as well as development of resistance.

## Introduction

Tuberculosis (TB) is a major global health concern and the increasing drug resistance makes TB-control even more demanding. Without adequate chemotherapy transmission of drug resistant TB will continue, and the suffering of the individual patients will increase. Concomitantly with the introduction of modern TB drugs over half a century ago, morbidity and mortality was dramatically reduced in countries like Sweden. Yet, TB remains a global epidemic, with one-third of the world's population infected, at least 9.9 million new active cases and 1.8 million deaths in 2008 [1]. In a recent survey, drug- and multidrug resistance (MDR, i.e. resistance to at least rifampicin (RIF) and isoniazid (INH)) was assessed, and the median global prevalence of drug resistance was estimated to be 20% [2]. The distribution of resistance varies substantially world wide with resistance to at least one drug ranging from 0% in some small Western European countries to 56.3% in Azerbaijan, and MDR TB ranging from 0% to 22.3%, again with highest frequency in Azerbaijan. An estimated 2.9% of all new TB cases worldwide have MDR-TB. Strains resistant also to the agents used in the therapy of MDR-TB such as the fluoroquinolone ofloxacin (OFL) and the injectable second-line drugs amikacin (AMI) were more recently described and named extensively drug resistant (XDR) [3]. A growing proportion of such XDR-TB cases will seriously obstruct TB control globally [4], [5].

**Figure 1:**
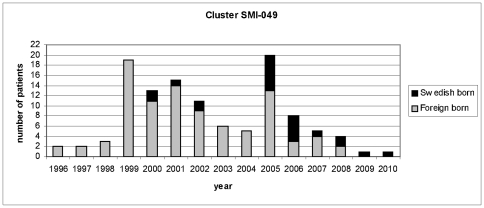
Number of new cases in cluster SMI-049 identified per year. The number of new cases belonging to the SMI-049 cluster is displayed for each year. Gray portions of bars represent foreign born patients, black portions of bars represent Swedish born patients.

In molecular epidemiological studies of drug resistant TB in Sweden a large outbreak of INH resistant TB was identified, mainly involving patients originating from the Horn of Africa. One exceptionally large cluster, SMI-049, was identified during the study period [6]. Beginning in 1996, two patients belonging to this cluster were identified per year, until 1999 when suddenly 19 new patients were identified. The identification of isolates belonging to cluster SMI-049 during several years and the large accumulation of new cases in 1999 indicated an active spread of TB in Sweden. For this reason a more thorough investigation was initiated in 2000 by the Swedish National Board of Health and Welfare. Yet, in spite of awareness of the outbreak, and strengthened contact investigations, new cases continued to appear (Fig. 1), first at a decreasing pace, then in 2005 again rapidly increasing. Up to date (November 2010) 115 patients infected with the cluster SMI-049 strain have been identified. Twenty-two patients belonging to cluster SMI-049 were Swedish born, and appeared in the later phase of the outbreak, indicating that the epidemic had gradually taken hold in the Swedish born population. Despite the extensive spread of this particular *M. tuberculosis* strain in the community it has stayed genetically unchanged since its discovery with regard to drug resistance, IS*6110* restriction fragment length polymorphism (RFLP)- and spoligotyping patterns. The apparent genetic stability together with the extensive spread of this strain in a low endemic country like Sweden led us to further characterize if the strain possess any particular genetic factors that could explain its evolutionary success and genetic stability.

Here we present a detailed analysis of the *M. tuberculosis* strain causing the cluster SMI-049 outbreak. The genomes of the isolate of the index case, and two SMI-049 isolates with isolation dates differing from the index strain by 9 years, were sequenced by massive parallel DNA sequencing using the 454-platform. Comparisons were made with previously sequenced *M. tuberculosis* strains and regions of known importance to antibiotic resistance and virulence were studied more closely. Phylogenetic studies and studies of *M. tuberculosis* sequence variation have previously mainly focused on unrelated isolates and not looked at whole genome sequences of clinically traceable strains over prolonged time. Interestingly, although the three isolates were obtained with 9 years in-between the only differences found on the whole genome scale were four single nucleotide polymorphisms (SNP) in one of the later isolated strains and a four amino-acids in-frame deletion in the second strain compared to the index strain. This shows that the cluster SMI-049 strain is exceptionally stable genetically and yet evolutionarily very successful and that clonally disseminated *M. tuberculosis* strains can stay virtually unchanged over many years and multiple transmission cycles.

## Methods

### Bacterial isolates

During the years 1994–2010, drug resistant (DR) *Mycobacterium tuberculosis* isolates were obtained from all the six Swedish TB laboratories in Gothenburg, Linköping, Malmö/Lund, Stockholm and Umeå. In Sweden, all isolates are tested for susceptibility to the first-line drugs isoniazid (INH), ethambutol (EMB) and rifampicin (RIF). During the major part of the study all isolates were also tested for susceptibility to streptomycin (SM), except for the years 2004–2010, when the Linköping and Stockholm laboratories stopped testing for SM-resistance, since SM no longer was used for treatment of TB patients in Sweden. All laboratories had taken part in the external quality assurance program for drug susceptibility testing of *M. tuberculosis* offered by the Swedish TB reference laboratory at the Swedish Institute for Infectious Disease Control (SMI), Solna, Sweden. The first isolate from each patient was included in the study.

### RFLP

The isolates were cultured on Löwenstein-Jensen medium. DNA was extracted by resuspending two blue loops of bacteria in 450 µL TE buffer pH 8.0 (10 mM Tris-HCl, 1 mM EDTA) and then heated at 80°C for 20 min. The cells were further freeze thawed twice and 30 µL of lysozyme (20 mg/mL) was added and incubated for 2 h at 37°C. Seventy µL of 10% sodium dodecyl sulfate (SDS) and 5 µL of proteinase K (10 mg/mL) were added to the lysate, vortexed and incubated for 10 min at 65°C followed by addition of 100 µL of 5 mol/L NaCl and 100 µL of 10% N-cetyl-N,N,N-trimethyl ammonium bromide. The tubes were vortexed until the solution was white and incubated for 10 min at 65°C. DNA was extracted by two chloroform-isoamyl alcohol (24∶1, vol/vol) treatments, precipitated by addition of cold isopropanol and the pellet was redissolved in TE buffer. RFLP typing was performed using the insertion sequence IS*6110* as probe and PvuII as restriction enzyme [7], [8]. Visual bands were analyzed using the BioNumerics software version 6.01 (Applied Maths, Kortrijk, Belgium). Strains with identical RFLP patterns (100% similarity) and consisting of five bands or more were judged to belong to a cluster. On the basis of the molecular sizes of the hybridizing fragments and the number of IS*6110* copies of each isolate, fingerprint patterns were compared by the un-weighted pair-group method of arithmetic averaging using the Jaccard coefficient. Dendrograms were constructed to show the degree of relatedness among strains according to a previously described algorithm [9] and similarity matrixes were generated to visualize the relatedness between the banding pattern of all isolates.

### Spoligotyping

All isolates were also characterized by spoligotyping [10]. The patterns obtained by spoligotyping were compared by visual examination and by sorting the results in BioNumerics. Spoligotypes in binary format were entered in the SITVIT2 database (Pasteur Institute of Guadeloupe), which is an updated version of the previously released SpolDB4 database [11], and were assigned to the major phylogenetic lineages according to signatures provided in SITVIT2, which defines 62 genetic lineages/sublineages of *M. tuberculosis* complex strains. At the time of the present study, SITVIT2 contained global genotyping information on about 73,000 *M. tuberculosis* complex clinical isolates from 160 countries of origin, with more than 3000 spoligo-international-types (SITs).

Three isolates, one from the index case, and two isolated nine years after the index case, were subjected to further analysis as follows:

### Drug susceptibility testing

Drug susceptibility testing for the drugs SM, EMB, RIF, AMI and OFL was performed at SMI by the radiometric BACTEC 460 assay according to the instructions of the manufacturer (Becton Dickinson Biosciences, Sparks, MD). The results were in accordance with those previously obtained at the regional laboratory.

### Sanger sequencing

Sanger DNA sequencing of the 980-bp fragment of *inhA* promoter region was performed using primers previously described [12], sequencing of the cluster SMI-049 specific variations in the *polA*, *PPE55* and *cyp138* genes was performed using the following primers: polA_Fwd 5′-CTGCAACTGGTCAGTGACGA, polA_Rev 5′-CGCAACACCCGAAACTCCA, PPE55_Fwd 5′-GTTGACATTGCCAGGGTTGA, PPE55_Fwd2 5′-GGATGTCGAACAGCGACATG, PPE55_Rev 5′- CAATGTCGGGTTCGGCAACT, PPE55_Rev2 5′-AACCTGGGCAACCACGTGTC, PPE55_Rev3 5′-ACCTGAATCCGCTGAACATC, CYP138_Fwd 5′-ATGGCGATCCCGACGTCTTC, CYP138_Rev 5′-CCAGCCTTTCACCCGAACTC. All Sanger sequencing was performed using The BigDye Terminator v3.1 Cycle Sequencing Kit (Applied Biosystems).

### Whole genome sequencing

Massive parallel sequencing using the 454-technology (Roche) was performed at the sequencing facility at the SMI. Chromosomal DNA of the S96-129, BTB05-552 and BTB05-559 isolates was extracted as for the RFLP analysis except for two additional extractions with chloroform-isoamylalcohol (24∶1, vol/vol). DNA was sequenced using the 454-FLX technology using the manufacturer's instructions (www.454.com) and data were processed with the accompanying software package. This generated 464949 and 502523 reads giving mean genome coverage of 23 and 24 times for S96-129 and BTB05-552, respectively. Isolate BTB05-559 was sequenced both using 454-FLX technology and also using the 454-Titanium upgrade generating a combined number of 373176 reads and a 32 times mean coverage. Gapped genome sequences of the three isolates have been deposited at GenBank as whole genome shotgun projects under accession numbers AEGB00000000 (S96-129), AEGC00000000 (BTB05-552) and AEGD00000000 (BTB05-559). The versions described in this paper are the first versions, xxxx01000000.

### Bioinformatics

Assembly and analysis of 454-sequencing data were performed with the CLC Genomics Workbench v3.7 (CLC Bio, Aarhus, Denmark). Assemblies were performed for each isolate individually and also as a combined assembly including all sequence data for a deeper coverage of the common features of the cluster SMI-049 isolates. Large sequence polymorphisms (LSP) between cluster SMI-049 isolates and H37Rv (NC_000962) were identified by detecting assembly positions with partially matching reads and the source of variation elucidated by a combination of *de novo* assembly of non-assembled reads, reference assembly of the SMI-049 data on the other three fully sequenced *M. tuberculosis* genomes H37Ra (NC_009525), CDC1551 (NC_002755) and F11 (NC_009565) and manual sequence identification of partially matching reads. SNP analysis was performed for each sequenced isolate after the large sequence polymorphisms had been introduced into a modified H37Rv-SMI049 reference genome to avoid false calls related to assembly problems at locations of large rearrangements. Analysis parameters were as follows: average base quality filter cutoff 15, central base quality filter cutoff 20, minimum variation frequency cutoff 75%, maximum variation (ploidy) 2, minimum sequence coverage 3. The SNP results for the three sequenced genomes were compared and all SNP calls that were not found in all three or had a variation frequency <90% were manually verified in the assemblies. Absence of sequence coverage at the position of an SNP in one or two of the sequences is denoted in Table S3.

### Growth rate and TNF induction in macrophages

Peripheral blood mononuclear cells (PBMCs) were isolated from the buffy coats of blood samples from healthy donors by density gradient centrifugation (Lymphoprep, Axis-Shield, Oslo, Norway). Monocytes were separated from PBMCs using CD14-positive magnetic beads (Miltenyi, Bergisch Gladbach, Germany) and were more than 98% pure. Monocytes were cultured in DMEM supplemented with 10% FCS, 1 mM sodium pyruvate, 50 U/ml penicillin, 50 µg/ml streptomycin (all from Gibco, Paisley, UK) and 40 ng/ml rhM-CSF (Peprotech, Rocky Hill, NJ, USA). On day 2 in culture, culture medium was exchanged, antibiotics withdrawn, and the cells were cultured for another three days to differentiate into macrophages.

Macrophages were seeded in 24-well plates (Nunc, Roskilde, Denmark) at a density of 5×10^5^/well or in Lab-Tek chamber slides (Nunc, Roskilde, Denmark) at a density of 3×10^5^/well. After 24 h cells were infected with mycobacteria at a multiplicity of infection of 1∶1. Three different mycobacterial strains were used: H37Rv (ATCC 27294), and the two cluster SMI-049 isolates S96-129 (isolate from index case) and BTB05-552 (late isolate). After 3 h, the cells were extensively washed to remove extracellular bacteria and were cultured for 6 days in DMEM.

On day 0, 1, 3 and 6 infected macrophages were lysed with 0.05% Triton X-100, lysates were plated on 7H11 Middlebrook agar and colony-forming units (cfu) were enumerated after 3–4 weeks. Macrophages in chamber slides were fixed with 4% paraformaldehyde and intracellular acid-fast bacteria were visualized using Kinyoun stain. Cells containing different numbers of bacteria were counted under 1000-fold magnification in consecutive microscope fields until a total of at least 10^3^ cells was attained and expressed as proportion of total number of infected cells.

TNF was quantified in culture supernatants harvested at different time points, using an ELISA according to the manufacturer's protocol (BD OptEIA Sets, BD Biosciences, San Diego, CA, USA).

## Results

### Cluster SMI-049

**Figure 2:**
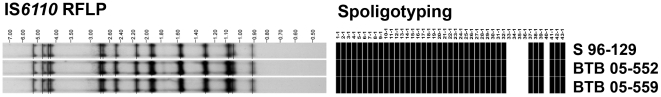
Stability in IS*6110* RFLP and spoligotyping patterns. IS*6110* RFLP and spoligotyping patterns of the three SMI-049 isolates S96-129, BTB 05-552 and BTB 05-559. Approximate molecular weights are indicated in kilo base pairs next to the RFLP gel picture. Spoligotype products 1–43 are depicted as black (positive) and white (negative) boxes.

During the years 1996-2010 115 cluster SMI-049 isolates from the same number of patients were isolated and characterized by drug susceptibility testing, IS*6110* RFLP, and spoligotyping. The cluster is defined by a low-level resistance to INH, a 14-band IS*6110* RFLP pattern and a spoligotyping pattern belonging to SIT52, of the T2 lineage, according to the SITVIT2 database (Fig. 2). Three isolates from three different patients were selected for further characterization. The first isolate (S96-129) from August 1996 was from the source case, a 19-year old male originating from Zaire (now Democratic Republic of the Congo). The second isolate (BTB05-552) from September 2005 was from a 28-year old Swedish woman and the third isolate (BTB05-559) from August 2005 was from a 6-year old Swedish born girl. In a timetable of cluster occurrence based on the estimated time for the development of first symptoms consistent with TB, isolate S96-129 is believed to be the source of the cluster (number 1), while BTB05-559 is number 88 and BTB05-552 is number 95 [13].

The cluster SMI-049 strain had been circulating in the population for about nine years between the time of diagnosis of the source case and the two other cases. In a chain of transmission the isolate BTB05-552 had been transmitted through at least three patients, more probably four patients, excluding the source case and case number 95 from which it was isolated, and the isolate BTB05-559 had been transmitted from the source case through two or three patients before infecting case number 88.

### Whole genome sequencing

Massive parallel sequencing of the genomes of *M. tuberculosis* isolates S96-129, BTB05-552 and BTB05-559 was performed using the Roche 454-sequencing technology. The mean sequence coverage of the genomes were 23, 24 and 32 times respectively and the completeness of the genome sequences were estimated to be 98% for all isolates. Zero coverage regions that could not be ascribed to deletions compared to the H37Rv reference genome were all located at loci with higher than average GC content, mainly in PE-PGRS genes (Polymorphic GC Rich Sequences), that are known to be difficult to sequence due to their high GC content [14], [15].

### Differences between isolates of cluster SMI-049

The three sequenced isolates had identical IS*6110* RFLP- and spoligotyping patterns like all 115 cluster SMI-049 isolates (Fig. 2). This pattern was very stable, in only one case a strain isolated from a patient belonging to the cluster in 2000 demonstrated a RFLP pattern with one extra band indicative of a new IS*6110* insertion event. The sequenced genomes each contain 18 copies of IS*6110* but the theoretical RFLP pattern fits well with the obtained 14-band pattern since some fragments have very similar sizes and will migrate together thus resulting in a lower than expected number of visible bands on the gel. However, the PvuII site in gene Rv2017 (with an IS*6110* inserted downstream) appears to be partially resistant to cleavage and only results in a weak band at 1735 bp. Instead, cleavage at the next following PvuII site results in a clear band at 2728 bp.

Comparison of the generated genome sequences reveals that the three sequenced isolates do not differ by any large sequence polymorphisms (LSPs). The only differences found were four SNPs in BTB05-559 and a four amino acids in-frame deletion in BTB05-552 compared to the index isolate S96-129. All differences were verified by manual Sanger sequencing. The SNPs in BTB05-559 consist of a synonymous C ->T transition at nucleotide position 579 in the *polA* gene (encoding DNA polymerase I) and three SNPs in the PPE55 gene that all result in amino acid changes (N1496Y, T1517A, I1520V). PPE55 belongs to a group of highly variable *M. tuberculosis* specific proteins with unknown function but that are highly immunogenic and secreted by the Type VII secretion pathway [16], [17]. The in-frame deletion in BTB05-552 removes amino acids 404-407 (out of 441) of the putative cytochrome P450 protein Cyp138.

### Comparison of cluster SMI-049 with H37Rv and other *M. tuberculosis* genomes

A total of 65 LSPs were identified between the SMI-049 isolates and H37Rv (33 insertions, 26 deletions, 6 combined insertion/deletions) with sizes of 9–6831 bp (Table S1). Twenty-five of these were associated with differences in the presence of the mobile insertion element IS*6110*. The second most common group of changes was copy number differences in regions of short intergenic repeats. The net difference in DNA content in the SMI-049 isolates is an addition of 9103 bp compared to H37Rv. In total, 33 of the polymorphisms were intergenic while 32 affected annotated genes. Twenty-four LSPs were unique to the SMI-049 cluster (i.e not found in any other *M. tuberculosis* genome at NCBI) and 12 of these affected annotated genes, 5 deletions and 7 IS*6110* insertions (Table 1). Of the five deletions, three affected PE or PPE-genes (PE-PGRS44, PPE38, PPE39, PPE57-59). Five large regions not present in H37Rv (1674 bp, 6836 bp, 953 bp, 5000 bp and 6771 bp in length, respectively (Table S1)) results in a total of 15 extra genes that are present in the SMI-049 strains but absent from H37Rv. However, all these regions are present in the CDC1551 strain and represent regions deleted in H37Rv. None of them have been reported to be important for virulence.

**Table 1:** SMI-049 specific large polymorphisms.

Reference position (H37Rv)	Allele variations	Size (bp)	Overlapping annotations	Change
164574-164585	Deletion	12	*Gene: Rv0136 cyp138.* 4 amino acids deletion specific for BTB05-552.	No frameshift
888927-889020	Deletion	94	*Intergenic:* Deletion of 94 bp compared to H37Rv	
1533690	Insertion	1413	*Intergenic:* IS6110 insertion not present in H37Rv	
1541951	Variation	44	*Intergenic:* IS6110 insertion site 44 bp downstream of corresponding IS6110 in H37Rv	
1987503	Insertion	1362	*Gene:* Rv1755 3′ part of *plcD*: Insertion of IS6110 199 bp upstream and in reversed orientation compared to H37Rv	Disruption
1987697-1989024	Inversion	1299	IS6110 in reversed orientation compared to H37Rv	
2038790-2039673	Insertion/deletion	1355/883	*Gene:* Rv1798 *lppT,* Putative lipoprotein: Truncated by IS6110 insertion and deletion of 883 bp	Disruption
2163462	Insertion	1497	*Gene:* Rv1917c *PPE34*: Insertion of IS6110 and 135 bp not present in H37Rv	Disruption
2245301	Insertion	1358	*Gene:* Rv2000, hypothetical protein: Insertion of IS6110 not present in H37Rv	Disruption
2367679	Insertion	1359	*Intergenic:* Insertion of IS6110 not present in H37Rv	
2634069-2635575	Deletion	1507	*Genes:* Rv2352c and Rv2353c, *PPE38* and *PPE39*: Deletion of 1507 bp compared to H37Rv. Smaller than DS9 [66]	Frameshift
2636931	Insertion	156	*Intergenic:* Insertion of 156 bp compared to H37Rv	
2902567-2904318	Insertion/deletion	122/1752	*Genes:* Rv2578c and Rv2579 *dhaA*, hypothetical protein and haloalkane dehalogenase: Deletion of 1752 bp + insertion of 122 bp	Disruption
2937529-2937530	Deletion	9	*Gene:* Rv2591 *PE-PGRS44*: Deletion of 9 bp compared to H37Rv	No frameshift
3125851	Insertion	1358	*Gene:* Rv2818c, hypothetical protein: Insertion of IS6110 not present in H37Rv	Disruption
3192248	Insertion	53	*Intergenic:* Insertion of 53 bp compared to H37Rv, intergenic repeat region	
3378898	Insertion	1359	*Gene:* Rv3019c, *esxR* secreted ESAT-6 like protein: Insertion of IS6110 not present in H37Rv	Disruption
3550843	Insertion	1357	*Gene:* Rv3183, possible transcriptional regulatory protein: Insertion of IS6110 not present in H37Rv	Disruption
3594319-3594430	Deletion	112	*Intergenic:* Deletion of 112 bp compared to H37Rv, intergenic repeat region	
3710316-3711736	Insertion/deletion	6771/1421	*Genes:* Rv3325 and Rv3326, IS6110 transposases: IS6110 replaced by 6771 bp insertion flanked by two IS6110 in reversed direction, similar to CDC1551	Deletion
3803868-3803918	Deletion	51	*Intergenic:* Deletion of 51 bp compared to H37Rv	
3842289-3847214	Deletion	4926	*Genes:* Deletion of Rv3425 to Rv3428c, *PPE57* and *PPE59* joined and IS1532 element lost through deletion of 4926 bp probably through homologous recombination via large repeat region. Similar to DS13 [66]	Deletion
4172770	Insertion	1358	*Intergenic:* IS6110 insertion not present in H37Rv	
4348818	Insertion	59	*Intergenic:* Insertion of 59 bp compared to H37Rv, intergenic repeat region	

SNP and small deletion/insertion polymorphism (DIP), 1–6 bp length, analyses were performed after the large sequence polymorphisms had been introduced into a modified H37Rv-SMI049 reference genome to avoid false calls related to assembly problems at locations of large rearrangements. The SNP and small DIP results were compared to the resequencing results of H37Rv [15], [18] and 90 SNPs and 11 DIPs resulting from identified errors in the H37Rv sequence were removed. In total, the cluster SMI-049 sequences differ from H37Rv by 777 SNPs (781 in BTB05-559, see above) and 44 small DIPs (Tables S2 and S3). Of the SNPs 73 occurred in intergenic regions and 724 in annotated genes. The majority of SNPs (56%) were non-synonymous in concordance with previous reports [18], [19], [20] illustrating the low level of purifying selection in *M. tuberculosis*. Premature stop-codons were found in six genes (Table 2). Of these only *bglS* (beta-glucosidase) and *lpdA* (lipoamide dehydrogenase) encode proteins with known functions and the premature stop codons occur at the very end of both genes.

**Table 2:** SMI-049 specific SNPs resulting in premature stop codons.

Reference position (H37Rv)	Reference	Allele variations	Overlapping annotations	Amino acid change
218204	C	T	*Gene:* Rv0186 *bglS*, beta-glucosidase	R646 Stp (691 aa)
281238	C	T	*Gene:* Rv0235c, probable transmembrane protein	W459 Stp (482 aa)
2129419	G	T	*Gene:* Rv1879, hypothetical protein	E15 Stp (378 aa)
2374245	C	T	*Gene:* Rv2114, hypothetical protein	Q138 Stp (207 aa)
3689523	G	T	*Gene:* Rv3303 *lpdA*, Lipoamide dehydrogenase	C472 Stp (493aa)
4351039	G	T	*Gene:* Rv3872 *PE35*	E99 Stp (99aa)

Twenty-one of the identified DIPs were specific for cluster SMI-049 and 16 of these resulted in frame shifts in annotated genes, five of which have predicted functions, *nanT*, *fadD35*, *ltp1*, *lppH* and *gidB* (Table 3). The first four are involved in lipid biosynthesis and transport and it is not clear what effect knock out of these genes may have on cell viability. However, GidB has recently been shown to be a highly conserved 7-methylguanosine (m^7^G) methyltransferase responsible for methylation of position G527 of the 16S rRNA [21]. All three strains sequenced have a deletion of G102 of the *gidB* gene. Loss of function of GidB is associated with low-level resistance to streptomycin [21], [22]. The remaining genes affected by DIPs either encoded hypothetical proteins (N = 4) or belong to PPE or PE-PGRS gene families (N = 6).

**Table 3:** SMI-049 specific small deletion and insertion polymorphisms (DIP).

Reference position (H37Rv)	Reference	Allele variations	Overlapping annotations	Change
33203	G	-	*Intergenic*	
336684	A	-	*Gene:* Rv0279c *PE_PGRS4*	Frameshift
336694	-	C	*Gene:* Rv0279c *PE_PGRS4*	Frameshift
671259	--	TG	*Gene:* Rv0577 *TB27.3*, hypothetical protein	Frameshift
789689	G	-	*Intergenic*	
839545	-	G	*Gene:* Rv0747 *PE_PGRS10*	Frameshift
1178876	AT	--	*Intergenic*	
1242287	G	-	*Gene:* Rv1118c, hypothetical protein	Frameshift
1280683	------	CGAAGT	*Gene:* Rv1153c *omt*, Putative O-methyltransferase	No frameshift
1537711	AC	--	*Intergenic*	
1982962	C	-	*Gene:* Rv1753c *PPE24*	Frameshift
2046760	C	-	*Gene:* Rv1803c *PE_PGRS32*	Frameshift
2149855	-	A	*Gene:* Rv1902c *nanT*, sialic acid-transport integral membrane protein	Frameshift
2821079	GG	--	*Gene:* Rv2505c *fadD35*, Putative acyl-CoA synthetase	Frameshift
3006362	G	-	*Gene:* Rv2689c, hypothetical protein	Frameshift
3100153	-	A	*Gene:* Rv2790c *ltp1*, Putative lipid -transfer protein	Frameshift
3742352	C	-	*Gene:* Rv3345c *PE_PGRS50*	Frameshift
4018414	-	A	*Gene:* Rv3576 *lppH*, Putative lipoprotein	Frameshift
4117362	C	-	*Gene:* Rv3677c, Putative hydrolase	Frameshift
4408101	C	-	*Gene:* Rv3919c *gidB*, 7-methylguanosine (m^7^G) methyltransferase	Frameshift

### Positional effects of IS*6110* insertions

*M. tuberculosis* contains several mobile insertion sequences (IS) [23]. IS*6110* is a member of the IS3-family and is found in almost all members of the *M. tuberculosis* complex in varying number of copies. Integration of IS*6110* can, depending on the site of insertion, result in phenotypic changes in the host bacterium, both detrimental as well as occasionally beneficial (Reviewed in [24]). To determine if any obvious effects of IS*6110* insertions could be predicted, we analyzed the insertions of IS*6110* in the SMI-049 cluster genomes in more detail. Eight IS*6110* insertions occur in annotated genes in SMI-049 and the affected open reading frames are most likely completely disrupted (Table 1). As expected, none of the affected genes are reported to be essential for *M. tuberculosis*. Notably, the PPE34 and *plcD* genes are the genes with most detected individual IS*6110* insertions in clinical isolates of *M. tuberculosis* [25] and disruption of these genes have been speculated to result in changes in the immunological interaction with the host [26], [27], [28], [29]. Of the remaining genes with insertions of IS*6110*, two have predicted functions, one in lipid biosynthesis (*lppT*) and one as a secreted ESAT-6-like gene (*esxR*) and four are hypothetical genes. Over-representation of IS*6110* insertion in hypothetical genes has been noted earlier [25].

Apart from disrupting genes, IS*6110* insertions have also been found to activate gene expression of nearby genes by an outward directed promoter (OP6110) at the 3′ end of the insertion element [30], [31], [32]. To assess if any IS*6110* insertions in the SMI-049 genomes could account for differences in virulence by the OP6110 promoter we mapped what genes were present nearby inserted IS*6110* (Table 4). Apart from the truncated genes that harbored an IS*6110,* and most likely are non-functional, five genes were found at a reasonable distance from an IS*6110* and oriented in the forward direction compared to OP6110, an IS1547 transposase, PPE36, PE27A, *moaC* (molybdenum cofactor biosynthesis protein C) and an oxidoreductase with unknown specificity. Of these genes, changed expression of PPE36 and PE27A might result in changes in the immunogenic response.

**Table 4:** Putative genes where IS*6110* insertions may alter promoter activity.

Position of OP6110 in SMI-049	Downstream gene	Function	Distance	Orientation of downstream gene	Comment
890769-890838	Rv0797	IS1547 Transposase	94 bp	Forward	
1537121-1537190	Rv1362c	Hyp. Prot., unknown function	398 bp	Reversed	
1545719-1545788c	Rv1368	*lprF*, probable lipoprotein	272 bp	Reversed	
1992219-1992288	Rv1755c	plcD 5′, Phospholipase C - truncated	86 bp	Reversed	IS6110 inserted into *plcD*
2000585-2000654	Rv1758	*cut1* 3′, Serine esterase, hydrolysis of cutin - truncated	66 bp	Forward	IS6110 inserted in *cut1, Non-functional * [*79*]
2051273-2051342	Rv1799	*lppt* 3′, probable lipoprotein - truncated	82 bp	Forward	IS6110 inserted into *lppT*
2175810-2175879c	Rv1917c	PPE34 3′, unknown function - truncated	81 bp	Forward	IS6110 inserted into PPE34
2260061-2260130	Rv2000	Hyp. Prot. 3′, unknown function - truncated	85 bp	Forward	IS6110 inserted into Rv2000
2280108-2280177	Rv2117	Transcriptional regulator protein	155 bp	Reversed	
2387585-2387654	Rv2108	PPE36, unknown function	119 bp	Forward	
2652563-2652632	Rv2356c	PPE40, unknown function	994 bp	Reversed	
3132243-3132312	Rv2813	Hyp. Prot., unknown function	1495 bp	Reversed	
3137724-3137793	Rv2818c	Hyp. Prot. 3′, unknown function - truncated	82 bp	Forward	IS6110 inserted into Rv2818c
3392133-3392202c	Rv3019c	*esxR* 5′, secreted ESAT-6 like protein - truncated	82 bp	Reversed	IS6110 inserted into *esxR*
	Rv3018A	PE27A, unknown function	564 bp	Forward	
3565496-3565565	Rv3182	Hyp. Prot., unknown function	210 bp	Reversed	
3723555-3723624c	Rv3324A	Pseudogen	84 bp	Forward	IS6110 inserted into Rv3324A
	Rv3324c	*moaC*, molybdenum cofactor biosynthesis protein C	194 bp	Forward	
3728662-3728732c	MT3429	Hyp. Prot., unknown function	79 bp	Reversed	Identical to MT3429 of CDC1551, not present in H37Rv
4184858-4184927	Rv3727	Oxidoreductase	269 bp	Forward	

### Growth rate and TNF induction in human macrophages

**Figure 3:**
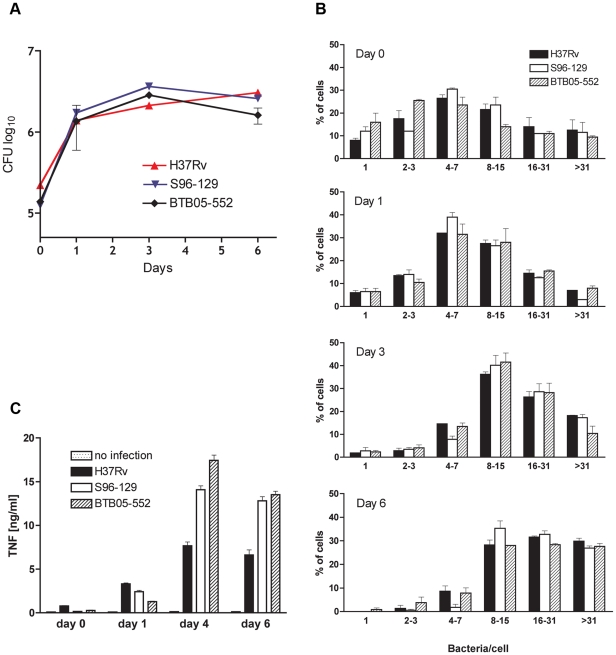
Virulence of SMI-049 isolates in human macrophages. Monocyte-derived human macrophages were infected with strain H37Rv or SMI-049 isolates S96-129 or BTB05-552, at multiplicity of infection 1∶1. Intracellular bacterial growth was determined by enumeration of cfu in plated macrophage lysates (A) or by estimation of proportion of cells containing varying amounts of bacteria (B) over time. TNF induction in infected macrophages was quantified by ELISA using culture supernatants harvested at different time points post-infection (C).

Cluster SMI-049 isolates S96-129 and BTB05-552 grew in macrophages as indicated by a successive increase in cfu in macrophage lysates (Fig. 3A) and an increase of proportion of cells containing higher numbers of bacteria over time (Fig. 3B). The growth rate of both SMI-049 cluster isolates was similar and comparable to that of the H37Rv laboratory strain until day 3. Of note, at day 6 less bacteria were recovered from cluster SMI-049-infected macrophages as compared with day 3, while macrophages infected with H37Rv showed further increment in cfu at that time point (Fig. 3A). Microscopic analysis of chamber slide cultures revealed that there was augmented death of macrophages infected with the SMI-049 isolates on day 6 as indicated by increased number of cells with fragmented cytoplasm and pycnotic nuclei; no increased cell death was observed in H37Rv-infected macrophages (not shown). Interestingly, from the day 4 post-infection SMI-049-infected macrophages produced much greater amounts of TNF than macrophages infected with H37Rv (Fig. 3C).

### Drug resistance genes

When tested by the BACTEC 460 reference method all three isolates were resistant to isoniazide (INH) at 0.2 mg/L. S96-129 was resistant to streptomycin (SM) at 4 mg/L whereas BTB05-552 and BTB05-559 were SM-susceptible to this concentration. All three isolates were susceptible to the first line drugs rifampicin (RIF), ethambutol (EMB) and pyrazinamide (PZA). These isolates were also susceptible to the second line drugs ofloxacin (OFL) (at 2 mg/L) and amikacin (AMI) (1 mg/L). Genes previously reported to be involved in resistance to antibiotics used to treat *M. tuberculosis* infections (*rpoB* – RIF; *katG*, *inhA*, *oxyR*-*ahpC, iniBAC* and *furA* – INH; *rrs*, *rpsL* and *gidB* – SM; *pncA* – PZA; *embB* – EMB; *gyrA* and *gyrB* –OFL) were specifically monitored for non-synonymous mutations that may change the tolerance to these antibiotics. Low-level INH resistance of cluster SMI-049 strains is explained by a -15 C to T transition in the promoter region of the *inhA* gene, which encodes an enoyl acyl carrier protein reductase involved in fatty acid synthesis [33]. This mutation has been reported as the second most common resistance mutation to INH and is most often associated with low (<10 ug/ml) level resistance [34], [35], [36], [37], [38]. Twenty-five randomly chosen isolates belonging to the SMI-049 cluster were also found to contain this mutation (data not shown). Only one other change was found in a gene previously reported to be involved in INH resistance, *iniA* Q394E, a substitution that has never been reported to alter drug susceptibility.

As noted above, all three isolates contain a deletion of G102 in the *gidB* gene that previously has been reported to result in low-level SM resistance [21], [22]. The low-level of resistance reported earlier for *gidB* mutants is just at the clinical break point and this may explain why S96-129 was found to be resistant while BTB05-552 and BTB05-559 were susceptible. No changes were found in *rrs* or *rpsL* genes that could explain the differences in SM susceptibility between the isolates.

The *gyrA* gene of all three isolates contain five non-synonymous mutations (E21Q, T80A, A90G, S95T and G668D) compared to H37Rv. Interestingly, change of alanine at position 90 to valine is commonly reported in quinolone resistant isolates [39], [40], [41] but change to a glycine in combination with the T80A mutation has instead been reported to result in hypersensitivity to quinolones [42]. This finding led us to determine the MIC for OFL more precisely by testing all three isolates at 2, 1, 0.5 and 0.25 mg/L. No growth was detected at any of these concentrations (only without antibiotics) and the MIC for OFL is therefore < = 0.125, well below the average wild-type distribution MIC of 0.5 [43]. This is interesting since the T80A mutation is a marker for the Uganda genotype [44]. The S95T mutation is known as a naturally occurring polymorphism among quinolone susceptible strains [45], [46] and E21Q and G668D are attributed to H37Rv specific differences and present in many other susceptible strains and should therefore not contribute any resistance.

### Phylogenetic background of SMI-049

Phylogenetically the SMI-049 isolates were of the modern principal genetic group 2 (PGG2) defined by *katG463* and *gyrA95* SNPs (katG463 CGG (Arg) and gyrA95 ACC (Thr)) [45]. By extended SNP analysis (Filliol 2006) they were found to belong to SNP cluster group 5 (SCG-5), which is predominant in Uganda [47]. The RFLP pattern of the cluster SMI-049 strain matches (92%) with a strain (BEA000007341) from Rwanda recorded in the international database of IS*6110* RFLP patterns maintained at RIVM, Bilthoven, the Netherlands, also indicating that the strain may have its origin in sub-Saharan Africa. According to the phylogeny in [20] and [44] SNPs of the three sequenced genomes placed the SMI-049 strain in the Lineage 4 (red) Europe and Americas lineages, with 2/3 matches to the “Uganda” cluster within the PGG2 group.

By spoligotyping all cluster SMI-049 isolates were of the T2 lineage, which is geographically linked to East Africa [11]. All isolates had the spoligotype international type SIT52. The sequenced isolates also lacked the region of difference 724 (RD724), a polymorphism that defines one major sub-lineage of *M. tuberculosis* commonly seen in the central African human host population, and which is common in isolates from Uganda [48], [49]. Together with a T2 specific spoligotype pattern [18] RD724 defines the “Ugandan genotype” [48], again supporting the concept that the cluster SMI-049 strain has its origin in Central/Eastern Africa.

## Discussion

The SMI-049 outbreak is one of the largest outbreaks of TB ever reported from low endemic countries. A few other outbreaks of this size have been reported from Denmark, England, The Canary Islands and The Netherlands [50], [51], [52], [53], the largest being two TB clusters comprising 184 and 272 cases respectively found over a ten years period in Denmark [54].

In spite of the long transmission time and many carriers of the cluster SMI-049 strain in the community, the IS*6110* RFLP pattern has stayed virtually unchanged and we found extremely few genomic changes (4 SNPs and one small deletion) between the three sequenced isolates that were isolated 9 years apart. This indicates that the SMI-049 lineage is genetically very stable both with respect to point mutations and to transpositional activity of insertion sequences, in contrast with reports of other *M. tuberculosis* lineages e.g. the Beijing lineage. Differences in the level of genetic variability have to be considered in view of the already very low genetic variability among members of the *M. tuberculosis* complex. Our genome sequences are lacking coverage of several PE-PGRS genes most likely as a result of sequencing bias due to high GC-content in those genes. It is therefore possible that there exists more variation between SMI-049 strains than we have detected in this study.

Niemann et al [18] found that two isolates of the Beijing lineage from an outbreak in Uzbekistan with identical IS*6110* RFLP fingerprinting pattern exhibited considerable genomic diversity (130 SNPs and one large deletion). The fact that strains with identical genotyping patterns can accumulate significant amounts of genetic diversity indicates that epidemiological links between strains with identical genotyping data can be more remote and are likely to represent older transmission events rather than cases of recent transmission among patients in one RFLP cluster. The two isolates studied by Niemann et al actually exhibited differences in MIRU/VNTR as well as in drug resistance patterns, indicating that they were genetically more remote than indicated by their identical RFLP pattern. In Estonia genetically closely related isolates of the Beijing lineage showed a range from full susceptibility to four-drug resistance, indicating that drug resistance had developed recently and independently in different clones of Beijing strains [55].

### Stability in RFLP

Yeh et al. found that about 30% of serial isolates of patients whose cultures spanned at least 90 days had changed IS*6110* RFLP patterns [56]. In contrast, here we found that the cluster SMI-049 strain has maintained the same stable IS*6110* RFLP profile over 9 years with only one isolate as an exception. This may indicate that the SMI-049 strain has a very low transposition activity of IS*6110* despite the presence of 18 copies in the genome. Low transposition activity is generally thought to occur in genomes with low number of IS*6110* copies (1–5 copies) [57]. However, transpositional activity has been linked to the transcriptional activity of the genomic location of IS*6110* elements with increased overall transpositional activity occurring when an IS*6110* element is inserted at a genomic position with strong transcriptional activity [58]. There also seems to be an upper limit of about 25 IS*6110* elements per genome, possibly regulated by a trans acting repressor expressed from each element that limits the transpositional activity more the more elements are present [24]. If any of these factors keep the IS*6110* pattern of the SMI-049 cluster so stable is hard to speculate upon.

### Stability in drug resistance

Although members of the *M. tuberculosis* complex have accumulated a very low general genetic variability since their last common ancestor, the modern use of anti-TB drugs presents a new selective pressure that can increase the genetic variability in genes that render the bacteria drug resistant by selecting those over their susceptible counterparts. In clinical isolates exposed to therapy, increased genetic variation should therefore be expected in drug resistance genes. However, some resistance mutations have been associated with fitness costs that might result in a reduced virulence/transmissibility (reviewed in [59]). All initial isolates of the 115 patients belonging to cluster SMI-049 were resistant to INH, but susceptible to EB and RIF. In only one instance a strain developed further resistance: in 2003 a patient was found to be infected with a strain belonging to the SMI-049 clone and resistant to INH, in 2004 another isolate with the same fingerprint from the same patient was in addition resistant to RIF, making the strain MDR [6]. This mutant variant with increased resistance did not spread further. It is intriguing that in spite of the long passage of this strain in the community there has been no further development of drug resistance. Resistance to INH has been coupled with various chromosomal mutations but those in *katG* and in the promoter regions of *inhA* have been most frequently associated with clinical isolates [60], [61]. The INH resistance of the cluster SMI-049 isolates was low level, with a MIC of 0.4 mg/L, which agrees with the fact that the isolates had the *inhA* promoter mutation. Strains with the *inhA* promoter mutation or the KatG S315T mutation were more likely to spread than strains with other mutations, suggesting that these mutations have a low fitness cost [62] which is in agreement with the stability in INH resistance of cluster SMI-049.

All three isolates contain the G102 deletion in *gidB* reported earlier to give low-level resistance to SM [21], [22]. This level of resistance is just at the clinical resistance break-point and this is probably why some isolates belonging to the cluster SMI-049 have been reported as resistant while most isolates are considered susceptible. We could not find any genomic difference between isolate S96-129 (SMR) and BTB05-552 (SMS) and BTB05-559 (SMS) that can explain this difference in resistance. Use of SM to treat infections of SMI-049 should therefore be avoided, especially since the *gidB* mutation has been reported to dramatically elevate the frequency of high-level mutations to SM in *M. tuberculosis* [22]. On the other hand, SMI-049 isolates are highly sensitive to quinolones and this group of antibiotics might therefore be useful for treatment.

### Effects of polymorphisms on evolutionary success and virulence

Large sequence polymorphisms (LSPs) such as deletions and insertions of IS-elements (mainly IS*6110*) represent a substantial source of genetic variation among *M. tuberculosis* lineages [63], [64]. How LSPs in the *M. tuberculosis* complex may affect the evolutionary success of certain lineages has been discussed frequently [64], [65], [66], [67]. There is increasing evidence that certain *M. tuberculosis* strains are particularly prone to disseminate and/or to cause disease [60], [68], [69], [70], [71]. There are also several examples of hypervirulence resulting from gene deletion in *M. tuberculosis* [72]. However, the potential pathogenic relevance of most specific regions of difference is unknown. Interestingly, the Beijing lineage that is causing concern due to its global distribution and its involvement in severe outbreaks does not seem to spread in Sweden [73]. Since the SMI-049 cluster strain has been extensively spread in Sweden, a country with low incidence of TB, questions have been raised whether it contains genotypic determinants that make it particularly prone to disseminate. The fact that transmission was very high during certain periods [13] around particular individuals might on the other hand indicate that part of the extensive transmission may be explained by socio-demographic factors.

SMI-049 does not contain any unique sequences not present in other *M. tuberculosis* strains. On the other hand there are several specific deletions and IS*6110* insertions not found elsewhere. The successful spread of SMI-049 and the fact that it is not attenuated in growth in human macrophages also shows that deletion of the genes absent from its genome compared to other sequenced strains is not likely to be detrimental to virulence. In fact our findings of the high TNF production in human macrophages infected by SMI-049 isolates *in vitro* paralleled by the augmented macrophage death support the concept of the sustained virulence of SMI-049; similar induction of increased TNF expression and cellular necrosis by virulent clinical strains, as compared to H37Rv, have recently been found in murine macrophages *in vitro* [74] and in a mouse model of TB infection [75].

In some instances deletion of genes has been associated with altered development of *M. tuberculosis* disease, e.g. Kong et al found a significant linkage between the deletions of RD105, RD181 and RD142 (which are markers for Beijing strains) and the occurrence of extrathoracic tuberculosis [28]. Many of the inactivating polymorphisms (deletions and IS*6110* insertions in genes) specific for the SMI-049 cluster have occurred in genes belonging to the PE and PPE-gene families (PE-PGRS44, PPE34, PPE38-39, PPE57-59). Since these gene classes are thought to play important roles for the bacterium by generating antigenic variation and immune evasion, variable occurrence of PE and PPE genes may change the interaction of the bacterium with its host and thus alter the progress of the disease.

SMI-049 isolates also have a unique IS*6110* inserted at a slightly different position in the *plcD* gene (Rv1755) compared to H37Rv. There are four phospholipase C genes (PLC) in *M. tuberculosis*, *plcABC* as an operon and *plcD* alone at a separate position in the genome. All four contribute to PLC activity and are involved in virulence although their exact role is not fully understood [76]. Expression of the *plc*-genes is upregulated after infection of macrophages and the quadruple knock out mutant is attenuated in growth in lungs and spleen of mice. Interruptions of *plc*-genes are frequent among clinical *M. tuberculosis* isolates and most frequent in *plcD* [27], [28], [77]. It is hypothesized that reduced PLC activity causes persistence of the bacterium inside macrophages and aid travel to distant organs. Alternatively, reduced activity limits release of arachidonic acid from the macrophage membrane and reduce immune response in the initial lung infection. It is therefore possible that the clinical presentation of the disease may be influenced by the genetic variability of the *plcD* region. It is not immediately obvious if any of the other genes with annotated functions affected by inactivating changes would mediate phenotypic changes in the cluster SMI-049 strains although *dhaA* (encoding a haloalkane dehalogenase) has been shown to be upregulated upon *in vitro* infection of a macrophage cell line [78].

In summary, we have found that the cluster SMI-049 strain that has caused a major outbreak of INH resistant TB in Sweden has been genetically extremely stable over extended time in the community and through many transmissions and has not developed resistance to the antibiotics used to treat patients infected with the strain. Even though we have found specific genetic features in this strain not found in other TB strains, the question why this particular strain of TB has been so successful in spreading both in the immigrant population where it originated and also in the Swedish born population remains to be answered.

## Supporting Information

Table S1 **Complete list of LSPs compared to H37Rv.**
(XLS)

Table S2 **Complete list of DIPs compared to H37Rv.**
(XLS)

Table S3 **Complete list of SNPs compared to H37Rv.**
(XLS)
